# Bottlebrush-architectured poly(ethylene glycol) as an efficient vector for RNA interference in vivo

**DOI:** 10.1126/sciadv.aav9322

**Published:** 2019-02-20

**Authors:** Dali Wang, Jiaqi Lin, Fei Jia, Xuyu Tan, Yuyan Wang, Xiaoya Sun, Xueyan Cao, Fangyuan Che, Hao Lu, Ximing Gao, Jackson Christopher Shimkonis, Zifiso Nyoni, Xueguang Lu, Ke Zhang

**Affiliations:** 1 Department of Chemistry and Chemical Biology, Northeastern University, Boston, MA 02115, USA.; 2 David H. Koch Institute for Integrative Cancer Research, Massachusetts Institute of Technology, Cambridge, MA 02139, USA.

## Abstract

Nonhepatic delivery of small interfering RNAs (siRNAs) remains a challenge for development of RNA interference–based therapeutics. We report a noncationic vector wherein linear poly(ethylene glycol) (PEG), a polymer generally considered as inert and safe biologically but ineffective as a vector, is transformed into a bottlebrush architecture. This topology provides covalently embedded siRNA with augmented nuclease stability and cellular uptake. Consisting almost entirely of PEG and siRNA, the conjugates exhibit a ~25-fold increase in blood elimination half-life and a ~19-fold increase in the area under the curve compared with unmodified siRNA. The improved pharmacokinetics results in greater tumor uptake and diminished liver capture. Despite the structural simplicity these conjugates efficiently knock down target genes in vivo without apparent toxic and immunogenic reactions. Given the benign biological nature of PEG and its widespread precedence in biopharmaceuticals, we anticipate the brush polymer–based technology to have a significant impact on siRNA therapeutics.

## Teaser

Bottlebrush-structured PEG substantially enhances the pharmacokinetics of conjugated siRNA and RNAi activity in vivo.

## INTRODUCTION

Small interfering RNAs (siRNAs) represent an important therapeutics paradigm for treating diseases spanning viral infections, hereditary disorders, and cancers (*1*–*3*). siRNAs function at a level beneath proteins in the central dogma of molecular biology and are thus promising alternatives to small-molecule inhibitors, especially for “undruggable” targets (*4*). Compared to antisense oligonucleotides (ASOs), RNA interference (RNAi) is believed to be a more efficient and robust technology for gene suppression, owing to the involvement of the catalytic RNA-induced silencing complex (RISC) (*5*, *6*). Considerable amounts of effort and capital have been invested in bringing siRNA therapeutics to the market. To date, at least 20 RNAi-based drug candidates have entered clinical trials worldwide, with several *N*-acetylgalactosamine–siRNA conjugates in late-stage trials in the United States. A lipid particle–formulated siRNA, patisiran, has recently been approved by the U.S. Food and Drug Administration for the treatment of polyneuropathy caused by hereditary transthyretin amyloidosis, becoming the long-awaited breakthrough in RNA therapeutics (*7*, *8*). Yet, despite major progress, clinical translation is mainly limited to diseases of/originating from the liver (*9*, *10*). A key challenge to realizing the broad potential of siRNA-based therapeutics involves the delivery of siRNAs to nonliver organs/tissues and across the plasma membrane of cells in vivo. Unmodified siRNA is digested by serum and cellular nucleases and is subject to rapid renal clearance because of its small size and thus has a half-life that is too short for clinical use (*11*). In addition, naked siRNA does not readily enter unperturbed cells even at millimolar concentrations owing to a combination of large size (~13 to 16 kDa), negative charge (~−35 mV), and hydrophilicity (*12*).

Methods to address these challenges include advanced delivery systems (e.g., polycationic polymers, lipids, proteins, and peptides) and, more popularly, direct chemical modifications of the oligonucleotide (*13*–*18*). The use of cationic species for electrostatic complexation/encapsulation with siRNA is efficient in facilitating cellular uptake and promoting RNAi activity in vitro but leads to greater potential for immunogenic responses, toxicity, and inconsistency in formulation (especially for complex delivery systems with multiple components) (*19*, *20*). Other than liposomes, carrier-based systems have not been proven relevant in a clinical setting (*8*). On the other hand, modification chemistries have greatly improved enzymatic stability, potency, and duration of RNAi in vivo, making it possible to bypass the need for complex carriers (*21*). In addition, certain modifications [e.g., phosphorothioates (PS)] result in nonspecific binding with serum proteins and thus avoidance of renal clearance and improved tissue uptake (*22*). However, the liver often remains the organ that receives most of the injected dose, followed by the kidney, bone marrow, adipocytes, and lymph nodes (*23*). Other possible drawbacks associated with chemical modifications may include liver and cardiovascular toxicity, prolonged blood coagulation times, thrombocytopenia, and reduced binding affinity for the target sequence (*24*). Therefore, to realize the full potential of siRNA drugs, a safe, simple, and efficient vector system that can address nonliver targets, ideally with (but not limited to) natural, unmodified oligonucleotides, is still very much sought after (*25*).

Here, we disclose a bottlebrush-architectured poly(ethylene glycol) (PEG)–siRNA conjugate as an in vivo vector to suppress the antiapoptotic B cell lymphoma 2 (Bcl-2) family proteins in a tumor xenograft mouse model. Termed pacRNA (polymer-assisted compaction of RNA), the conjugate consists of approximately two strands of phosphodiester (PO) siRNA covalently attached to the backbone of the bottlebrush polymer bearing ~30 PEG side chains (10 kDa each) via a bioreductively cleavable disulfide linkage or a noncleavable bond. PEG can create a large hydration shell to shield covalently tethered species and has widespread precedence in pharmaceutical development with more than a dozen PEGylated pharmaceuticals on the market (*26*, *27*). The spatial congestion of the bottlebrush polymer resulting from the densely spaced PEG side chains provides steric shielding for the siRNA much more effectively than linear or slightly branched PEG, which circumvents many of the side effects associated with specific and nonspecific nucleic acid–protein interactions. Lacking these interactions, the pacRNAs exhibit considerably prolonged blood circulation times (~25× higher elimination half-life compared with unmodified siRNA) and elevated drug plasma availability [~19× greater area under the curve (AUC_∞_)], which allows the pacRNA to target mouse tumor xenografts passively via the enhanced permeability and retention (EPR) effect (*28*). We further show that it is possible to use native siRNA (without further chemical modifications or formulation beyond PEGylation) to regulate Bcl-2 expression and reduce tumor growth in vivo. Collectively, we demonstrate that PEG, a biologically benign polymer normally lacking vector-like properties, can be transformed into an efficient in vivo vector simply by altering its architecture. The finding should open up tremendous opportunities for developing new siRNA-based therapeutics for nonhepatic targets.

## RESULTS

### Preparation and physicochemical characterization of pacRNA

**Fig. 1:**
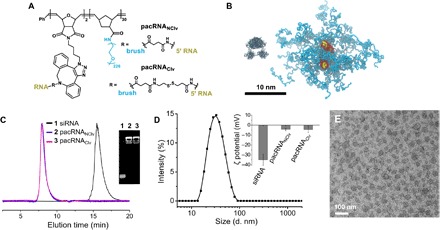
Physical characterization of pacRNA. (**A**) Chemical structure of pacRNA. (**B**) A coarse-grained molecular dynamics simulation of the pacDNA (1-μs simulation with explicit water using the MARTINI force field). A crystal structure of *Escherichia coli.* RNase III is placed next to the pacRNA for size comparison. (**C**) Aqueous GPC chromatograms and agarose gel electrophoresis (1%; inset) of pacRNAs and free siRNA. (**D**) DLS intensity-average hydrodynamic diameter distribution of pacRNA_Clv_. Inset, ζ potential measurements of siRNA and pacRNAs in Nanopure water. (**E**) TEM image of pacRNA_Clv_, negatively stained with 2% uranyl acetate.

To achieve optimal shielding of the siRNA and avoid renal clearance, the brush polymer must have side chains that are sufficiently dense (>25 repeating units in the backbone) and long (>10 kDa) and have an overall molecular mass above the renal filtration limit (~60 kDa) (*29*, *30*). Thus, we have designed the pacRNA with 30 PEG_10 kDa_ repeating units and approximately two strands of siRNA (Fig. 1, A and B). The brush polymer was synthesized via sequential ring-opening metathesis polymerization of 7-oxanorbornenyl bromide (ONBr) and norbornenyl PEG (NPEG), to yield a diblock architecture (pONBr_5_-*b*-pNPEG_30_), followed by azide substitution of the bromide (scheme S1). The first, oligomeric block serves as a reactive region for RNA conjugation, while the second, longer PEG block creates the steric congestion needed to shield the RNA. The guide strand of the siRNA was synthesized with a 5′ amine group, which was used to react with a cleavable linker (dibenzocyclooctyne-disulfide-*N*-hydroxysuccinimidyl ester) or a noncleavable linker (dibenzocyclooctyne-*N*-hydroxysuccinimidyl ester). The resulting products were purified by reverse-phase high-performance liquid chromatography and their structures were confirmed by MALDI-TOF (matrix-assisted laser desorption/ionization–time-of-flight mass spectrometry) (fig. S1). Subsequently, the dibenzocyclooctyne-terminated guide RNA strands were coupled to the brush polymer via copper-free click chemistry (*31*, *32*), followed by hybridization with the passenger strand, to yield both the bioreductively cleavable conjugate (pacRNA_Clv_) and the noncleavable version (pacRNA_NClv_). The molecular mass of the final conjugate is 330 kDa, with a polydispersity index (PDI) of 1.2 (fig. S2). The successful synthesis of the pacRNA is corroborated by aqueous gel permeation chromatography (GPC) and agarose gel electrophoresis (Fig. 1C and fig. S2). Note that the upward gel migration of pacRNA is a consequence of the transient interaction of PEG with passing cations during electrophoresis and not because of a net positive charge (*33*). ζ potential measurements indicate that the pacRNAs have a slight negative charge (−4.5 mV) in Nanopure water, which is below that of free siRNA (~−35 mV; Fig. 1D, inset). The pacRNAs exhibit a spherical morphology, with a dry-state diameter of ~30 nm, as evidenced by transmission electron microscopy (TEM) (Fig. 1E). The size is consistent with coarse-grained molecular dynamics simulation (Fig. 1B) and with dynamic light scattering (DLS) measurements, which show a *Z* average hydrodynamic diameter of 32 ± 2 nm and a PDI of 0.17 (Fig. 1D).

**Fig. 2:**
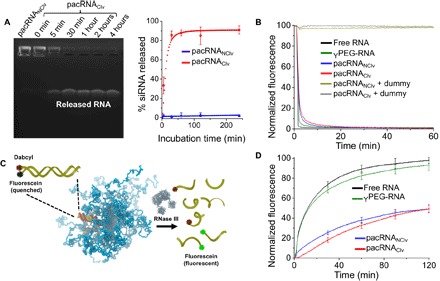
Reductive release, hybridization, and enzymatic degradation of pacRNA. (**A**) Agarose gel electrophoresis showing the reductive release of siRNA from pacRNA_Clv_ in the presence of 10 mM DTT as a function of time; release profile by gel band densitometry analysis is shown on the right. (**B**) Schematics of enzymatic digestion kinetics assay based on fluorophore- and quencher-tagged RNA. (**C** and **D**) RNA hybridization and RNase III degradation kinetics for pacRNAs, a 40-kDa Y-shaped PEG-RNA conjugate, and free RNA.

The redox responsiveness of pacRNA_Clv_ was tested by treatment with 10 mM dithiolthreitol (DTT) in phosphate-buffered saline (PBS), a condition often used to mimic the reductive intracellular environment. A time-course release profile was obtained by gel densitometry analysis of the released siRNA (Fig. 2A), which shows that ~80% of the siRNA was released after 30 min. In contrast, the stable pacRNA_NClv_ resulted in no release of the siRNA throughout the reaction. With a few exceptions, the cytoplasmic environment of tumor cells maintains a higher concentration of glutathione (GSH) than disease-free cells and much higher than typical serum levels (~1 mM) (*34*). Therefore, the difference in GSH concentration may be a contributing mechanism for passive tumor targeting, in addition to the EPR effect.

We next studied whether the pacRNAs remain capable of hybridization with a complementary (sense) sequence, and whether the elevated PEG density allows the pacRNA to resist nuclease degradation. Hybridization and nuclease degradation are monitored by a fluorescence quenching assay, in which a quencher (dabcyl)–modified sense strand is mixed with fluorescein-labeled pacRNA containing the antisense strand. Upon hybridization, the fluorophore-quencher pair is brought to proximity, resulting in a reduction in the fluorescence signals. Degradation, on the other hand, results in the release of the fluorophore and an increase in fluorescent signal. The rates of fluorescence loss and gain are therefore indicators of the hybridization and nuclease degradation kinetics, respectively. As shown in Fig. 2B, both pacRNAs hybridized with the sense strand rapidly, with negligible difference compared with free RNA. When a scrambled sequence was added, there was no change in the fluorescent signals, which rules out nonspecific interactions. To examine the extent of nuclease resistance, we added RNase (ribonuclease) III [an endoribonuclease specific for double-stranded RNA (dsRNA)] to prehybridized fluorophore/quencher–bearing duplexes (0.4 U/ml). The pacRNAs exhibited substantially prolonged nuclease half-lives (*t*_0.5_ = 120 ± 4 min) compared with that of naked dsRNA (*t*_0.5_ = 11.0 ± 0.5 min). In contrast, a Y-shaped PEG (40 kDa)–siRNA conjugate (an architectural control) only exhibited slightly increased half-life (*t*_0.5_ = 13 ± 2.5 min), confirming that the densely grafted architecture of brush PEG is critical to promoting steric selectivity (Fig. 2, C and D).

### Cellular uptake, intracellular release, gene regulation, and in vitro biocompatibility

**Fig. 3:**
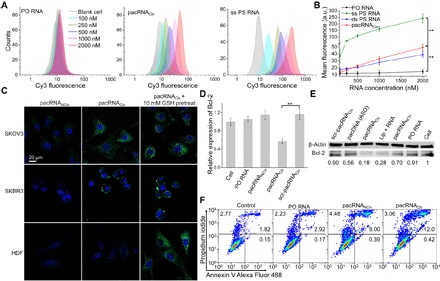
In vitro cellular uptake and gene silencing using pacRNA. (**A**) Flow cytometry measurement (total cell count: 10,000) of SKOV3 cells treated with PO RNA, PS RNA, and pacRNA (100 to 2000 nM) for 4 hours. (**B**) Mean fluorescence intensity of cells treated with free RNA (PS and PO) and pacRNA_Clv_. a.u., arbitrary units. (**C**) Confocal microscopy images showing intracellular GSH-triggered release of siRNA from pacRNA_Clv_ in high-GSH cells (SKOV3 and SKBR3). Fluorescence is turned on when the fluorophore (fluorescein)–labeled siRNA is released from the quencher (dabcyl)–labeled brush polymer. Controls include low-GSH HDF cells (negative) and cells pretreated with 10 mM GSH-OEt (positive). (**D**) qRT-PCR measurement (mean ± SD; *n* = 3) of Bcl-2 transcript levels in SKOV3 cells treated with pacRNAs, free siRNA, and pacRNA_Clv_ containing a scrambled control sequence. (**E**) Bcl-2 protein levels characterized by Western blotting. (**F**) Cell apoptosis following sample treatment determined by annexin V and propidium iodide (PI) staining. Early apoptotic, late apoptotic, and necrotic cell populations (%) are shown in the lower right, upper right, and upper left quadrants, respectively. Results are representatives of three independent flow cytometry measurements. **P < 0.01 (two-tailed *t* test).

Naked PO nucleic acids undergo insignificant cellular uptake, which may occur via non–receptor-mediated endocytosis (i.e., fluid phase or adsorptive) (*35*, *36*). The somewhat “nonsticky” character of nucleic acids is one of the reasons necessitating polycationic carrier systems, which enhance cellular delivery and subsequent release from endosomes to the cytosol (*37*). Certain chemically modified nucleic acids such as PS, on the other hand, are promiscuous binders to serum, cell membrane, and intracellular proteins, resulting in not only the ultimate high uptake by cells/tissues but also limited circulation in the blood and increased potential for off-target effects (*22*). Therefore, we hypothesize that the ability to moderately enhance cellular uptake above that of free nucleic acid but still maintain an overall biological stealth character is an important requirement for a long-circulating vector. To examine the cellular uptake of the pacRNAs, we treated human ovarian carcinoma cells (SKOV3) and breast adenocarcinoma cells (SKBR3) for 4 hours with 0.1 to 2 μM cyanine 3 (Cy3)–labeled conjugates, free PO RNA, and a full PS version of the RNA [both single stranded (ss) and double stranded (ds)], and subsequently analyzed them by confocal fluorescence microscopy and flow cytometry. Consistent with previous studies, free PO siRNA exhibited negligible cell uptake. The pacRNA_Clv_, on the other hand, showed 10× (SKOV3) and 22× (SKBR3) faster cell uptake compared to free RNA (Fig. 3, A and B, and figs. S3 to S5). While the increases are significant, the uptake for ss PS RNA was 60 to 82× faster than free RNA, which is comparable to the level of Lipofectamine-assisted delivery. The uptake for ds PS RNA was substantially slower than that of the ss version (~^1^/_10_ of the rate), suggesting that the conformational freedom of ss PS RNA is important for its interaction with serum and membrane proteins and subsequent endocytosis. Confocal microscopy confirmed that, in all cases, the RNA or conjugates were internalized by the cell, as opposed to being surface-associated (figs. S3 to S5). We speculate that the pacRNA enters the cell via a non–receptor-mediated endocytotic process similar to that of naked PO RNA and free PEG (*38*), but the near-neutral surface charge of the conjugate improves the transient particle-cell interactions, thereby allowing for moderately improved uptake (*35*). There has been evidence indicating that the negative charge is the main reason for the low uptake of nucleic acids, as opposed to the hydrophilicity, which is consistent with our study (*36*).

To investigate whether the internalized pacRNA can release the siRNA payload in tumor cells, we designed a fluorescence off-on assay using fluorescein-labeled siRNA conjugated to the quencher (dabcyl)–modified bottlebrush polymer. The turn-on of fluorescence is indicative of siRNA release (Fig. 3C). When tumor cells (SKOV3 and SKBR3) were treated with pacRNA_Clv_, apparent fluorescence was observed by confocal microscopy, from mainly within compartmentalized vesicles, while only very weak signals were detected in normal cells [primary human dermal fibroblasts (HDF)] under identical imaging settings. The result agrees with previous findings that the levels of intracellular GSH in certain tumor cells including SKOV3 and SKBR3 are several times higher than that in normal cells and that the disulfide bond–reducing activity can occur within the endocytotic vesicles (*39*). In contrast, the stable pacRNA_NClv_ exhibited little fluorescence in both tumor and normal cells, indicating that the kinetics of intracellular enzymatic cleavage of the siRNA is much slower compared with the bioreductive cleavage of the disulfide bond. For control, all cells were preincubated with 10 mM GSH monoester (GSH-OEt) for 2 hours to enhance the intracellular GSH level, followed by incubation with pacRNA_Clv_. With the pretreatment, fluorescence signals in all cells appear higher, and HDF cells in particular showed similar fluorescence levels to cancer cells. This observation provides direct evidence of bioreductive siRNA release from the pacRNA_Clv_, which is important for subsequent siRNA loading into the RISC (a critical step in RNAi).

To evaluate the RNAi efficacy in vitro, we incubated SKOV3 cells with pacRNA containing a Bcl-2 siRNA sequence (table S1) and controls. Quantitative real-time polymerase chain reaction (qRT-PCR) showed that the Bcl-2 transcript level was reduced by 43% with pacRNA_Clv_ treatment but remained nearly unchanged with the scrambled pacRNA_Clv_ and free siRNA controls (Fig. 3D). Western blotting confirmed that the pacRNA_Clv_ was the most effective in RNAi, with ∼82% knockdown by band densitometry analysis, more effective than Lipofectamine-based transfection (72%). In comparison, an ASO-based pacDNA control and the noncleavable pacRNA_NClv_ reduced Bcl-2 expression by 44 and 30%, respectively (Fig. 3E and fig. S6A). The same experiments were also performed with SKBR3 cells, and a similar general trend was observed (fig. S6, B and C). Decreased Bcl-2 expression has been observed to correlate with an increase in the levels of executioner proteins (Bax and Bak) in the cytoplasm, which facilitate the permeabilization of the mitochondria outer membrane and activation of the intrinsic apoptotic pathway (*40*). We monitored the apoptosis of SKOV3 cells treated with pacRNAs and controls by fluorescein isothiocyanate–annexin V/PI staining. Treatment with pacRNA_Clv_ resulted in the highest induction of apoptosis (12.42%), with most of the apoptosing cells in the late phase. The stable conjugate, pacRNA_NClv_, also resulted in 8.39% of apoptotic cells (8.00% in the late phase), despite lower potency in gene silencing (Fig. 3F and fig. S6D). In contrast, treatment with free siRNA did not result in an appreciable number of apoptotic cells beyond the untreated cells. Collectively, these functional readouts strongly imply that pacRNA_Clv_ is able to achieve some level of cytosolic delivery of siRNA for engagement with the RISC. It is possible that a fraction of the endocytosed pacRNA_Clv_ exits the endosome before bioreductive cleavage of the disulfide bond, thereby delivering the siRNA to the cytosol. This hypothesis is supported by the fact that a stable pacDNA_NClv_ structure carrying an ASO sequence exhibits antisense gene-silencing activity without the ASO being released, which indicates that the bottlebrush structure is able to gain access to the cytosol. However, the extent and mechanism of endosomal escape remains to be fully investigated.

**Fig. 4:**
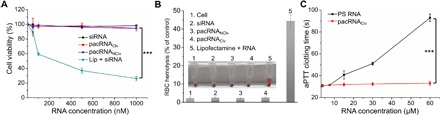
Cytotoxicity and blood compatibility of pacRNA. (**A**) Cell viability of SKOV3 cells treated with pacRNAs and controls. (**B**) Hemolysis of human blood (type O^+^) treated with pacRNAs and controls, as determined by spectrophotometric measurement of hemoglobin present in the supernatant of centrifuged RBC suspensions. The %RBC hemolysis is defined as the percentage of hemoglobin present in the supernatant compared with the total hemoglobin released by Triton X-100 treatment. Inset, photograph of centrifuged RBC suspensions. (**C**) Activated partial thromboplastin times of plasma treated with pacRNA or ss PS RNA at equal RNA concentrations. ***P < 0.001 (two-tailed *t* test).

A key advantage of the pacRNA is the realization of vector-like properties using almost only PEG and native RNA, relying instead on the steric selectivity resulting from the unique architecture of the bottlebrush polymer. Hence, we anticipate the pacRNAs to be free of the typical drawbacks associated with cationic carriers and chemically modified nucleic acids. 3-(4,5-Dimethyl-thiazol-2-yl)-2,5-diphenyl tetrazolium bromide cytotoxicity assay indicated no toxicity for pacRNA-treated SKOV3 cells up to 1 μM RNA (highest concentration tested), whereas Lipofectamine (cationic lipids) exhibited a half maximal inhibitory concentration of 270 nM, as expected from a typical polycationic carrier (Fig. 4A). In addition, high positive ζ potential synthetic vectors and surfactant-like carriers (e.g., liposomes, micelles, etc.) often display varying levels of blood incompatibility, e.g., aggregation of erythrocytes, hemolytic activity, etc. The pacRNA, being slightly negatively charged and completely hydrophilic, exhibited no detectable hemolysis, as estimated by measuring the amount of the hemoglobin released from red blood cells (RBCs) treated with an equivalent of 1 μM RNA under physiological conditions. For comparison, Triton X-100 (a nonionic PEG-based surfactant; 1% v/v) and Lipofectamine resulted in 100 and 44% hemolysis, respectively (Fig. 4B). Another benefit of the pacRNA is the ability to inhibit protein association in a complex biological environment, which reduces the likelihood of unwanted side effects. As a demonstration, we examined the anticoagulation properties of pacRNA versus PS RNA in human plasma (Fig. 4C). PS sequences exhibit strong nonspecific interactions with serum proteins such as thrombin, resulting in pronounced prolongation of activated partial thromboplastin time (aPTT), which leads to increased risk of bleeding in patients (*41*). The PS RNA (60 μM) showed marked anticoagulation activities, raising the aPTT by ~3×. In contrast, the pacRNAs at equal RNA concentrations resulted in only slight changes (<5%) in clotting times, indicating that the pacRNA does not disrupt the normal functions of proteins involved in the blood coagulation pathway. Together, these data suggest that the pacRNAs can be a safe and effective siRNA vector for in vivo RNAi.

### Pharmacokinetics, biodistribution, in vivo antitumor efficacy, and safety

**Fig. 5:**
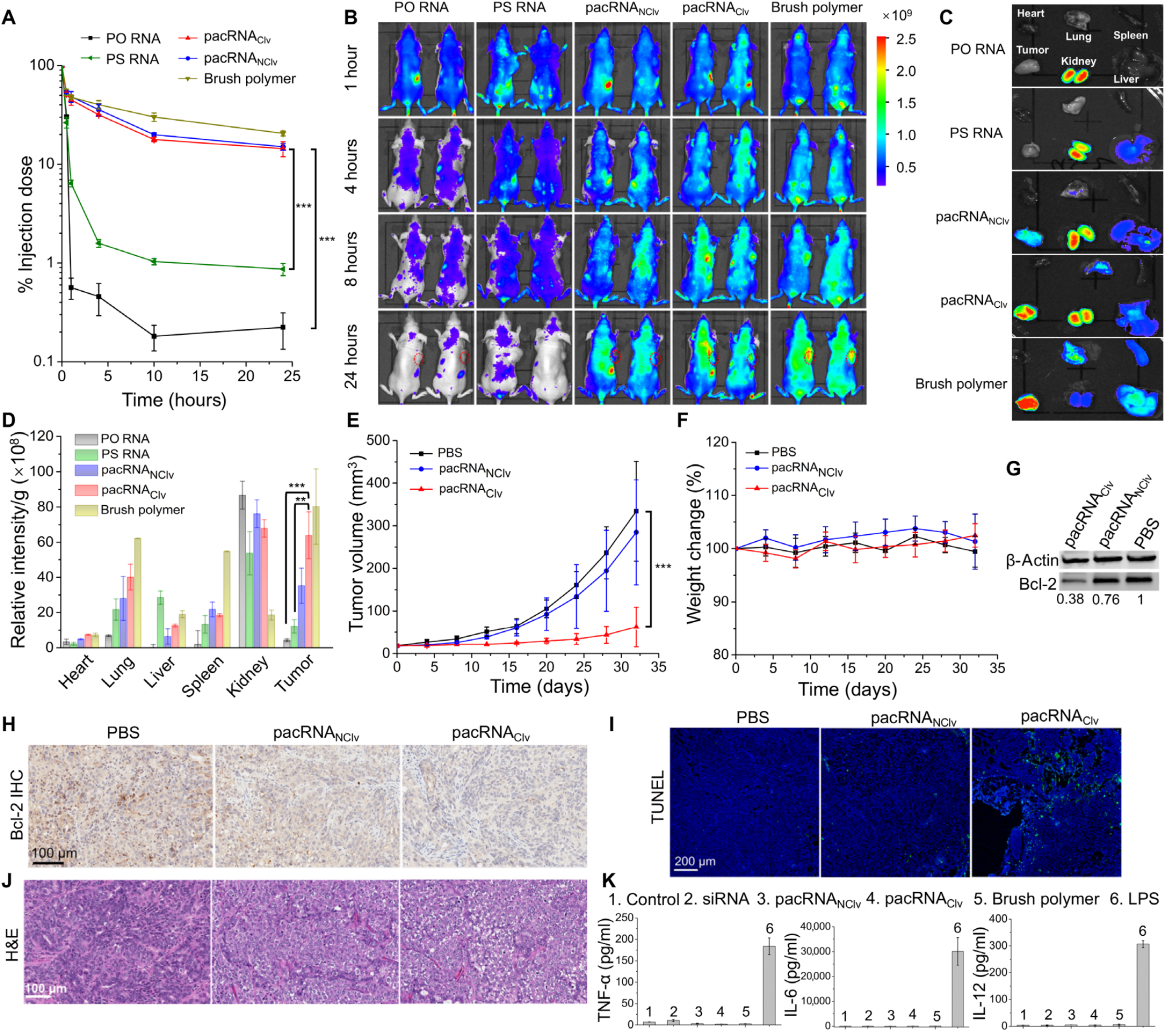
Pharmacokinetics, biodistribution, efficacy, and safety assessment in vivo. (**A**) Plasma pharmacokinetics of PO RNA, PS RNA, pacRNA, and the brush polymer in C57BL/6 mice. (**B**) Fluorescence imaging of BALB/c nude mice bearing a human ovarian SKOV3 xenograft following intravenous injection of Cy5-labeled siRNA and pacRNAs, or the Cy5.5-labeled brush polymer. The red circle indicates the location of tumors. (**C**) Ex vivo imaging of tumors and other major organs 24 hours after injection. (**D**) Biodistribution determined by quantitative analysis of the fluorescence signals of ex vivo tumors and major organs. (**E**) Tumor volume changes in the course of 32 days with intravenous administration of PBS, pacRNA_Clv_, and pacRNA_NClv_ at equivalent siRNA doses every fourth day (treatment started on day 0). (**F**) Body weight changes of tumor-bearing mice during the course of treatment. (**G**) Western blot of Bcl-2 in homogenized tumor tissues 96 hours after the last treatment. (**H** to **J**) Histological studies showing reduced Bcl-2 expression [immunohistochemistry staining (IHC)] (H), increased cell apoptosis (TUNEL) (I), and histologic apoptosis hallmarks (H&E staining) (J) in SKOV3 tumors treated with pacRNA_Clv_ compared with control groups. (**K**) Cytokine levels (TNF-α, IL-6, and IL-12) in the serum in C57BL/6 mice after 8 hours of treatment with pacRNAs and controls. **P < 0.01, ***P < 0.001 (two-tailed *t* test).

One main mechanism for anticancer nanomedicine systems to reach the pathological site is through blood circulation and extravasation via compromised vasculature, followed by intratumoral retention (*42*). Therefore, the dosage requirements for achieving high enough tumor concentration of the nanomedicine strongly depend on the longevity of the drug in blood circulation. To evaluate the plasma pharmacokinetics of the pacRNA, we injected immunocompetent C57BL/6 mice in the tail vein with free pacRNAs (both pacRNA_NClv_ and pacRNA_Clv_), the brush polymer lacking an RNA component, and naked siRNA (both PO or PS) at equal RNA/polymer concentrations. Blood samples at various predetermined time points up to 24 hours were collected and analyzed (Fig. 5A). All samples rapidly distributed into tissues with distribution half-lives (*t*_1/2α_) ≤30 min, but bottlebrush-containing samples showed much longer elimination half-lives (*t*_1/2β_ = ~13 to 20 hours) compared with free PS siRNA (*t*_1/2β_ = 53 min) or PO siRNA (*t*_1/2β_ = 34 min). There were also vast differences in plasma concentration. At 1 hour after injection, there was only ~0.6% of the injected free PO siRNA remaining in the plasma. While the PS siRNA exhibited significantly longer blood retention (~7.0% at 1 hour) likely due to binding with plasma proteins and therefore reduction in the glomerular filtration and urinary excretion, the bottlebrush polymers were much more effective in blood retention, with >47% of the injected dose remaining in circulation at 1 hour and 20.6% at 24 hours. The pacRNAs exhibited similarly improved blood availability (~15% at 24 hours), suggesting that the brush polymer can evade renal clearance and impart its biological stealth character to the siRNA. The vast differences in blood concentration and elimination rates result in substantially elevated blood availability of the pacRNAs compared with naked siRNA, with or without the PS backbone [AUC_pacRNA(Clv),∞_/AUC_PO RNA,∞_ = ~19]. All pharmacokinetic parameters are summarized in table S2.

The improved pharmacokinetics of pacRNA greatly enhanced siRNA accumulation at subcutaneously inoculated SKOV3 tumor sites in BALB/c mice, likely via the EPR effect. Fluorescence imaging of both live animals and the dissected organs 24 hours after injection suggests that free PO siRNA was quickly and primarily cleared by the kidney, while the PS siRNA rapidly accumulated in the liver, as well as the kidney (Fig. 5, B and C). Tumor uptake was minor or unobservable for the PS or PO siRNA-treated mice, respectively. Notably, the bottlebrush polymer exhibited the highest abundance in the tumor, followed by the lung, spleen, and liver (Fig. 5D), suggesting effective tumor targeting. The tumor levels for pacRNA_Clv_ and pacRNA_NClv_ are 80 and 44% relative to the free brush, respectively, indicating that the siRNA is not completely shielded by the brush. Once cleaved, the fragments are subject to rapid renal clearance. The ratio of tumor versus kidney uptake (as determined by mean fluorescence per gram of tissue) is 4.3 for the free brush, 1.0 for pacRNA_Clv_, and 0.5 for pacRNA_NClv_. Notably, the fluorescent tag is located at the outer periphery of the siRNA component on the pacRNA, and therefore cleavage at any position would cause the release of the fluorophore. It has not escaped our notice that the pacRNA_Clv_, having an additional bioreductive cleavage mechanism compared with the enzyme-only pacRNA_NClv_, accumulates more in the tumor despite a greater chance of releasing the siRNA. We attribute this phenomenon to the different locations where the cleavage may happen. It is possible that the pacRNA_NClv_ primarily liberates fragments of the siRNA due to enzymatic cleavage while in blood circulation. The pacRNA_Clv_, on the other hand, releases the siRNA more rapidly via bioreductive cleavage at the tumor site, which overpowers enzymatic cleavage, causing additional tumoral retention of the siRNA. To further examine tumor penetration depths, we sliced tumors, stained them with DAPI (4′,6-diamidino-2-phenylindole), and imaged them by confocal fluorescence microscopy (fig. S7). Appreciable fluorescence was detected in the tumors for pacRNA-treated and brush polymer–treated mice, while free siRNA resulted in no detectable fluorescence presumably due to poor pharmacokinetics. Notably, RNA fluorescence can be observed throughout the tumor section, including the center (~2.5-mm depth), suggesting enhanced penetration.

To further investigate the antitumor efficacy of the pacRNAs, an SKOV3 xenograft model was established in athymic nude mice (BALB/c). When the xenograft reaches a volume of ca. 20 mm^3^, pacRNA_Clv_, pacRNA_NClv,_ or vehicle (PBS) was administered in the tail vein (0.5 μmol/kg) once every fourth day for a total of eight doses. By day 32, the average tumor volume in the vehicle-treated group had progressed to ∼334 mm^3^, while the pacRNA_NClv_- and pacRNA_Clv_-treated groups exhibited reduced tumor growth, averaging ∼284 and ∼62 mm^3^, respectively (Fig. 5E). Suppression of Bcl-2 has been shown to inhibit tumor growth in several mouse xenograft models including H146 and H1963 (small cell lung cancer), and SKOV3; increase sensitivity to anticancer drugs; and enhance survival (*43*, *44*). Mice receiving pacRNA_Clv_ showed the most appreciable down-regulation of Bcl-2 protein expression (62% knockdown) following treatment, as evidenced by Western blotting of homogenized tumor tissues (Fig. 5G). Immunohistostaining of tumor sections using anti–Bcl-2 antibodies revealed substantially reduced Bcl-2 immunoreactivity (brown deposits) in pacRNA_Clv_-treated tumors compared with pacRNA_NClv_- and vehicle-treated groups (Fig. 5H). Furthermore, terminal deoxynucleotidyl transferase–mediated deoxyuridine triphosphate nick end labeling (TUNEL) showed a much greater abundance of apoptotic cells for the pacRNA_Clv_-treated group compared to control groups (Fig. 5I). Histologic apoptosis hallmarks (shrinkage of nucleus, formation of apoptotic bodies, and decreased cellularity) were observed for the pacRNA_Clv_-treated group by hematoxylin and eosin (H&E) staining of tumor sections, while the vehicle-treated group exhibited typical tumor histology, including pleomorphism and high nuclei-to-cytoplasm ratio (Fig. 5J). Together, these results strongly support the pacRNA_Clv_ as an effective long-circulating vector for in vivo gene silencing.

Next, we performed an initial in vivo safety analysis of the pacRNA. Because of the benign chemical composition of the pacRNA, typical toxic and immunogenic side effects are not expected. Throughout the 32-day treatment period, mice body weight for all treatment and control groups remained constant, and no obvious changes in behavior (refusal to eat, startle response, etc.) were observed (Fig. 5F). Furthermore, H&E staining of tissues obtained from major organs (including heart, spleen, liver, lung, and kidneys) in pacRNA-treated groups showed no histological variations from those of the vehicle-treated control group (fig. S8). Nonspecific immunoactivation by RNA, pacRNAs, and the bottlebrush polymer was also investigated in C57BL/6 mice after intravenous injection by monitoring the release of cytokines related to the innate and adaptive immunity (Fig. 5K). Enzyme-linked immunosorbent assay (ELISA) showed no changes in the levels of tumor necrosis factor–α (TNF-α), interleukin-6 (IL-6), and IL-12 in the serum of mice. In contrast, lipopolysaccharide, a positive control, exhibited very strong activation of the three cytokines. These data indicate that the pacRNA is not prone to acute toxic and immunogenic responses in mice and point to a generally safe agent for in vivo use. However, because of the widespread use of PEG in nonpharmaceutical applications, certain individuals may develop preexisting anti-PEG immunity, which may require prescreening to rule out should the pacRNA or similar PEG-based structures move forward in clinical translation.

## DISCUSSION

An ideal siRNA vector for in vivo use must fulfill at least two main purposes safely. First, it must facilitate cellular uptake and enhance RNAi activity. Second, it must direct the siRNA toward the target tissue effectively. The pacRNA achieves the first goal by masking the charge of the oligonucleotide and thereby moderately increases cellular uptake. The lack of high degrees of nonspecific cellular interactions and internalization is beneficial because targeting ligands may be incorporated to enhance specific cell targeting and because such interactions may lead to poorer pharmacokinetics and clearance by the mononuclear phagocyte system. In addition, the pacRNA is a noncationic vector, stabilizing the RNA by steric shielding instead of electrostatic complexation, which annuls the potential negative effects associated with polycationic agents. The stability of pacRNA makes it more likely to survive the digestive endosomal environment and successfully deliver a fraction of the intact conjugate to the cytosol, where release of the siRNA can function in RNAi.

The bottlebrush architecture also assists the siRNA in realizing the second major purpose of a vector by substantially increasing the molecular weight of the siRNA. The large size allows the siRNA to effectively escape the glomerular filtration without needing to bind with serum proteins, which enables prolonged blood retention and pronounced passive targeting of tumor tissues via the EPR effect. In addition, the steric shielding limits potential specific/nonspecific interactions between the siRNA and blood, cell membrane, and intracellular proteins, which should reduce the likelihood for side effects including coagulopathy and inadvertent activation of the immune system. Past efforts to PEGylate oligonucleotides failed to achieve these substantial improvements in biopharmaceutical properties because of the use of mainly linear or slightly branched PEG, which result in insufficient local density surrounding the oligonucleotide payload. All observed physiochemical and biopharmaceutical enhancements over naked siRNA are realized using primarily PEG (other than the brush backbone), which should promote the overall safety of the vector. Being a molecular entity with a well-defined structure as opposed to a supramolecular assembly of various components is another advantage for the pacRNA over heterogeneous vectors, which pose additional challenges in large-scale manufacturing and batch-to-batch consistency.

In conclusion, by simply altering the architecture of PEG, we have converted a safe but non–vector-like polymer into a promising class of noncationic agent for in vivo gene silencing. The densely packed PEG side chains of the bottlebrush protect the siRNA from degrading enzymes and other proteins, facilitate cellular uptake, and improve pharmacokinetics and tumor targeting, which result in outstanding therapeutic efficacy. We anticipate that the realization of the first architecture-based in vivo vector will drive the emergence of future systems with varying chemical compositions (to bypass potential anti-PEG immunity, further improve properties, etc.) and architectures (to increase local density, achieve molecularly purity, etc.) for nonhepatic RNAi. The pacRNA reported here serves as the basis for developing more customized therapeutic delivery vehicles based on materials that can be safely used in vivo.

## MATERIALS AND METHODS

### Intracellular siRNA release

Confocal microscopy was used to characterize the intracellular siRNA release using SKOV3, SKBR3, and HDF cells. Cells were seeded in 24-well plates at 1.0 × 10^5^ cells per well and cultured in Dulbecco’s modified Eagle’s medium (DMEM) (SKOV3 and HDF) or RPMI (SKBR3) medium at 37°C with 5% CO_2_ for 12 hours. For positive control, cells were pretreated with 10 mM GSH-OEt for 2 hours, followed by washing 3× using PBS. The off-on fluorescent probes in PBS solution were added to the wells at a final RNA concentration of 1 μM. The cells were further incubated for 4 hours, before washing with PBS and staining with Hoechst 33342. Confocal fluorescence images were captured using an LSM 700 confocal laser scanning microscope. Imaging settings were kept identical for all samples.

### Silencing of Bcl-2 using pacRNA in vitro

Cells were plated at a density of 2.0 × 10^5^ cells per well in 24-well plates in DMEM (SKOV3) or RPMI (SKBR3) medium and cultured overnight at 37°C with 5% CO_2_. Thereafter, samples and controls (1 μM equivalent RNA) in serum-free medium were added to the wells and incubated with the cells for 6 hours, before fresh medium was used to replace the incubation mixture. Cells were cultured for another 42 hours (qRT-PCR) or 66 hours (Western blot) before transcript and protein levels, respectively, were measured. For Bcl-2 mRNA transcript levels, a two-step qRT-PCR method was used. The total RNA was extracted using TRIzol following manufacturer-suggested protocols, and the RNA concentration was determined using a NanoDrop2000 spectrophotometer (Thermo Fisher Scientific, MA, USA). Total RNA [0.2 optical density unit at 260 nm (OD_260_)] was reverse-transcribed using SuperScript III (Thermo Fisher Scientific, MA, USA) at 42°C for 30 min and then at 85°C for 5 min. The obtained complementary DNA (cDNA) was amplified with SsoAdvanced Universal SYBR Green Supermix on a Bio-Rad CFX96 Touch qRT-PCR system (Bio-Rad Laboratories Inc., CA, USA). Results were normalized to glyceraldehyde phosphate dehydrogenase (GAPDH) expression. Each data point is the mean of three replicate experiments, each performed in triplicate. The following were the primer sequences used: Bcl-2, 5′-CAT GAG GCT CAG CCC CAG AAC-3′ (forward) and 5′-AGT CAA TCC CTT TGG TGC TCA C-3′ (reverse); GAPDH, 5′-TGC ACC ACC AAC TGT TTA GC-3′ (forward) and 5′-GGC ATG GAC TGT GGT CAT GAG-3′ (reverse).

For Western blotting, cells were harvested following incubation, and whole-cell lysates were collected in 100 μl of radioimmunoprecipitation assay cell lysis buffer with 1 mM phenylmethylsulfonylfluoride (Cell Signaling Technology Inc., MA, USA) following the manufacturer’s protocol. Protein content in the extracts was quantified using a bicinchoninic acid protein assay kit (Thermo Fisher Scientific, MA, USA). Equal amounts of proteins (30 μg per lane) were separated on 4 to 20% gradient SDS–polyacrylamide gel electrophoresis and electro-transferred to nitrocellulose membrane. The membranes were then blocked with 3% bovine serum albumin in tris-buffered saline supplemented with 0.05% Tween 20 and further incubated with β-actin (1:1000 dilution) and Bcl-2 (1:1000 dilution) primary and secondary antibodies (5000:1 dilution; Invitrogen Co., CA, USA). Protein bands were visualized by chemiluminescence using the ECL Western Blotting Substrate (Thermo Fisher Scientific, MA, USA). Each data point is the mean of three replicate experiments.

### Cell apoptosis

SKOV3 cells were treated with samples/controls in the same fashion as those used in the Western blotting study. For apoptosis analysis, both floating and attached cells were harvested, rinsed 3× with cold PBS, stained with Alexa Fluor 488 annexin V and PI, and analyzed by flow cytometry to identify apoptotic cells.

### Biodistribution and tumor targeting

Animal protocols were approved by the Institutional Animal Care and Use Committee of Northeastern University and carried out in accordance with the approved guidelines. For biodistribution, fluorescence whole-animal and ex vivo tissue imaging was used to monitor the Cy5.5-labeled brush polymer and the Cy5-labeled RNA component of the pacRNAs. To establish an SKOV3 tumor model, 2.0 × 10^6^ cells suspended in PBS were inoculated in female BALB/c nude mice by subcutaneous injection. When the tumor volume reached approximately 200 to 300 mm^3^, the mice were administered with samples and controls at equal RNA concentrations (500 nmol/kg; free brush concentration equals that of the pacRNA; *n* = 4) via tail vein injection and scanned at 1, 4, 8 and 24 hours using an IVIS Lumina II imaging system (Caliper Life Sciences Inc. MA, USA). After 24 hours, mice were euthanized using CO_2_, and major organs and the tumor were removed for biodistribution analysis. The amount of sample retention in the tissue was normalized to the weight of the corresponding tissues. For the analysis of tumor penetration depth, tumors were immediately frozen in optimal cutting temperature compound (Fisher Scientific Inc., USA) 24 hours after injection. The frozen tumor tissues were cut into 8-μm-thick sections using a cryostat, stained with DAPI, and imaged by confocal microscopy (Carl Zeiss Ltd., Cambridge, UK).

### Pharmacokinetics

Immunocompetent mice (C57BL/6) were used to examine the pharmacokinetics of free RNA (ds, both PS and PO), pacRNAs, and the free brush polymer lacking an RNA component. Mice were randomly divided into five groups (*n* = 4). Samples were intravenously administered via the tail vein at equal RNA concentrations (500 nmol/kg; free brush concentration equals that of the pacRNA). The blood samples (50 μl) were collected from the submandibular vein at varying time points (30 min and 2, 4, 10, and 24 hours) using BD Vacutainer blood collection tubes with sodium heparin. Heparinized plasma was obtained by centrifugation at 3000 rpm for 15 min, aliquoted into a 96-well plate, and measured for fluorescence intensity on BioTek Synergy HT (BioTek Instruments Inc., VT, USA). The amounts of RNA and brush polymers in the blood samples were estimated using standard curves established for each sample in freshly collected plasma. To establish the standard curves, samples of known quantities were incubated with freshly collected plasma for 1 hour at room temperature before fluorescence was measured.

### In vivo antitumor efficacy

The SKOV3 tumors were established in BALB/c female nude mice as described above. The tumors were inspected by visual observation and palpation. When the tumor volume reached approximately 20 mm^3^, the mice were randomly divided into three groups (*n* = 4): (i) vehicle (PBS) only, (ii) pacRNA_NClv_, and (iii) pacRNA_Clv_. Samples were injected via the tail vein once every fourth day for 28 days. The volume of tumors and weight of mice were recorded before every treatment and the fourth day after the last treatment. Antitumor activity was evaluated in terms of tumor size (*V* = 0.5*ab*^2^; *a*, long diameter; *b*, short diameter) by measuring two orthogonal diameters at various time points.

### In vivo immune response

Innate immune responses following the injection of samples were evaluated using C57BL/6 mice. Samples and a positive control (lipopolysaccharide, 15 or 150 μg per mouse; *n* = 4) were intravenously administered via the tail vein at equal RNA concentrations (500 nmol/kg; free brush concentration equals that of the pacRNA). Eight hours after administration, blood samples were collected for cytokine analysis using ELISA kits (R&D Systems Inc., MN, USA).

### Statistics

All experiments were repeated at least three times unless otherwise indicated. Data are presented as means ± SD. Statistical significance was evaluated by using two-tailed *t* test when only two groups were compared. If more than two groups were compared, evaluation of significance was performed using one-way analysis of variance (ANOVA) followed by Bonferroni’s post hoc test. Statistical significance was set at *P* < 0.01 or *P* < 0.001.

## Supplementary Material

http://advances.sciencemag.org/cgi/content/full/5/2/eaav9322/DC1

## SUPPLEMENTARY MATERIALS

Supplementary material for this article is available at http://advances.sciencemag.org/cgi/content/full/5/2/eaav9322/DC1 Supplementary Materials and Methods Scheme S1. Synthesis of pacRNA. Fig. S1. Synthetic scheme and characterization of dibenzocyclooctyne-modified RNA. Fig. S2. Additional characterization of brush polymers and pacRNAs. Fig. S3. Cellular uptake of PO siRNA, PS RNA, and pacRNA in SKOV3 cells. Fig. S4. Cellular uptake of PO siRNA, PS RNA (ss and ds), and pacRNA in SKBR3 cells. Fig. S5. Representative confocal images of SKBR3 cells treated with Cy3-labeled ss PS RNA or ds PS RNA for 4 h. Fig. S6. Bcl-2 down-regulation and cell apoptosis induced by pacRNA. Fig. S7. Fluorescence images of SKOV3 tumor cryosections following intravenous injections of siRNA, pacRNAs, and brush polymers. Fig. S8. Microscopic images of H&E-stained sections of various organs from mice after a 32-day treatment period with pacRNAs and PBS showing no apparent histological anomalies. Table S1. Oligonucleotide sequences. Table S2. Plasma pharmacokinetic parameters in C57BL/6 mice. References (*45*–*51*)
