# Supplementary material for: Bottlebrush-architectured poly(ethylene glycol) as an efficient vector for RNA interference in vivo
Source: Sci Adv. 2019 Feb 20;5(2):eaav9322. doi: 10.1126/sciadv.aav9322 (PMC6382396; doi:10.1126/sciadv.aav9322)
Supplement: http://advances.sciencemag.org/cgi/content/full/5/2/eaav9322/DC1 [file supp_5_2_eaav9322__index.html]

Science Advances | Science Advances

## Supplementary Materials

**This PDF file includes:**

- Supplementary Materials and Methods
- Scheme S1. Synthesis of pacRNA.
- Fig. S1. Synthetic scheme and characterization of dibenzocyclooctyne-modified RNA.
- Fig. S2. Additional characterization of brush polymers and pacRNAs.
- Fig. S3. Cellular uptake of PO siRNA, PS RNA, and pacRNA in SKOV3 cells.
- Fig. S4. Cellular uptake of PO siRNA, PS RNA (ss and ds), and pacRNA in SKBR3 cells.
- Fig. S5. Representative confocal images of SKBR3 cells treated with Cy3-labeled ss PS RNA or ds PS RNA for 4 h.
- Fig. S6. Bcl-2 down-regulation and cell apoptosis induced by pacRNA.
- Fig. S7. Fluorescence images of SKOV3 tumor cryosections following intravenous injections of siRNA, pacRNAs, and brush polymers.
- Fig. S8. Microscopic images of H&E-stained sections of various organs from mice after a 32-day treatment period with pacRNAs and PBS showing no apparent histological anomalies.
- Table S1. Oligonucleotide sequences.
- Table S2. Plasma pharmacokinetic parameters in C57BL/6 mice.
- References (*45*–*51*)

Download PDF

**Files in this Data Supplement:**

- Adobe PDF - aav9322\_SM.pdf
