# Supplementary material for: Bottlebrush-architectured poly(ethylene glycol) as an efficient vector for RNA interference in vivo
Source: Sci Adv. 2019 Feb 20;5(2):eaav9322. doi: 10.1126/sciadv.aav9322 (PMC6382396; doi:10.1126/sciadv.aav9322)
Supplement: http://advances.sciencemag.org/cgi/content/full/5/2/eaav9322/DC1 [file aav9322_SM.pdf]

Science Advances
AAAS

advances.sciencemag.org/cgi/content/full/5/2/eaav9322/DC1

Supplementary Materials for

Bottlebrush-architectured poly(ethylene glycol) as an efficient vector for RNA interference in vivo

Dali Wang, Jiaqi Lin, Fei Jia, Xuyu Tan, Yuyan Wang, Xiaoya Sun, Xueyan Cao, Fangyuan Che, Hao Lu, Ximing Gao, Jackson Christopher Shimkonis, Zifiso Nyoni, Xueguang Lu*, Ke Zhang*

*Corresponding author. Email: xueguang@mit.edu (X.L.); k.zhang@northeastern.edu (K.Z.)

Published 20 February 2019, *Sci. Adv.* **5**, eaav9322 (2019)
DOI: 10.1126/sciadv.aav9322

This PDF file includes:

Supplementary Materials and Methods
Scheme S1. Synthesis of pacRNA.
Fig. S1. Synthetic scheme and characterization of dibenzocyclooctyne-modified RNA.
Fig. S2. Additional characterization of brush polymers and pacRNAs.
Fig. S3. Cellular uptake of PO siRNA, PS RNA, and pacRNA in SKOV3 cells.
Fig. S4. Cellular uptake of PO siRNA, PS RNA (ss and ds), and pacRNA in SKBR3 cells.
Fig. S5. Representative confocal images of SKBR3 cells treated with Cy3-labeled ss PS RNA or ds PS RNA for 4 h.
Fig. S6. Bcl-2 down-regulation and cell apoptosis induced by pacRNA.
Fig. S7. Fluorescence images of SKOV3 tumor cryosections following intravenous injections of siRNA, pacRNAs, and brush polymers.
Fig. S8. Microscopic images of H&E-stained sections of various organs from mice after a 32-day treatment period with pacRNAs and PBS showing no apparent histological anomalies.
Table S1. Oligonucleotide sequences.
Table S2. Plasma pharmacokinetic parameters in C57BL/6 mice.
References (*45–51*)

Supplementary Materials and Methods

Materials. ω-Amine PEG methyl ether (M_n=10 kDa, PDI=1.05) was purchased from JenKem Technology, USA. Dibenzocyclooctyne-SS-*N*-hydroxysuccinimidyl ester (DBCO-SS-NHS) and dibenzocyclooctyne-*N*-hydroxysuccinimidyl ester (DBCO-NHS) were purchased from Sigma-Aldrich Co., USA. Phosphoramidites and supplies for DNA synthesis were obtained from Glen Research Co., USA. Human SKBR3 and SKOV3 cancer cell lines were purchased from American Type Culture Collection (Rockville, MD, USA). All other materials were obtained from Fisher Scientific Inc., USA, or VWR International LLC., USA, and used as received unless otherwise indicated.

Instrumentation. ^{1}H and ^{13}C nuclear magnetic resonance (NMR) spectra were recorded on a Varian 400 MHz NMR spectrometer (Varian Inc., CA, USA). MALDI-TOF MS measurements were performed on a Bruker Microflex LT mass spectrometer (Bruker Daltonics Inc., MA, USA). UV-Vis data were obtained on a Cary 4000 UV-Vis spectrophotometer (Varian Inc., CA, USA). Fluorescence spectroscopy was carried out on a Cary Eclipse fluorescence spectrophotometer (Varian Inc., CA, USA). Reverse-phase HPLC was performed on a Waters (Waters Co., MA, USA) Breeze 2 HPLC system coupled to a Symmetry® C18 3.5 μm, 4.6×75 mm reverse phase column and a 2998 PDA detector, using TEAA buffer (0.1 M) and HPLC-grade acetonitrile as mobile phases. *N*, *N*-dimethylformamide (DMF) GPC was performed on a TOSOH EcoSEC HLC-8320 GPC system (Tokyo, Japan) equipped with a TSKGel GMH_{HR}-H, 7.8×300 mm column and RI/UV-Vis detectors. HPLC-grade DMF with 0.04 M LiBr was used as the mobile phase, and samples were run at a flow rate of 0.5 mL/min. GPC calibration was based on polystyrene standards (706 kDa, 96.4 kDa, 5970 Da, 500 Da). Aqueous GPC measurements were carried out on a Waters Breeze 2 GPC system equipped with an Ultrahydrogel™ 500, 7.8×300 mm column and a 2998 PDA detector. Sodium nitrate solution (0.1 M) was used as the eluent running at a flow rate of 0.8 mL/min. Gel electrophoresis was performed using 0.5% agarose gel in 0.5× tris/borate/EDTA (TBE) buffer with a running voltage of 100 V. Gel images were acquired on an Alpha Innotech Fluorochem Q imager. DLS and ζ potential data were recorded on a Malvern Zetasizer Nano-ZSP (Malvern, UK). TEM samples were imaged on a JEOL JEM 1010 electron microscope with an accelerating voltage of 80 kV.

Oligonucleotide synthesis. Oligonucleotides (RNA and DNA, both PO and PS versions) were synthesized on a Model 391 DNA synthesizer (Applied Biosystems, Inc., Foster City, CA) using standard solid-phase phosphoramidite methodology. All RNA strands were cleaved from the CPG support using ammonium hydroxide/40% aqueous methylamine (1:1) solution at 65°C for 10 minutes. The 2'-*O*-triisopropylsilyloxymethyl (TOM) protecting group was removed by treatment with triethylamine trihydroflouride (TEA·3HF) in dimethylsulfoxide (DMSO) at 65°C for 2.5 h. DNA were cleaved from the CPG support using aqueous ammonium hydroxide (28-30% NH_3 basis) at room temperature for 24 h. Both DNA and RNA were purified by reverse-phase HPLC liquid chromatography. The successful syntheses of all DNA and RNA sequences were verified by MALDI-TOF MS.

Synthesis of norbornenyl bromide (2, Scheme S1). Maleimide (4.0 g, 41.2 mmol) and furan (3.08 g, 45.2 mmol) were dissolved in ethyl acetate (35 mL). The reaction mixture was refluxed for 4 h, and a white precipitate was observed. The solids were isolated by filtration, washed with diethyl ether, and

dried under vacuum to afford the product, 7-oxabicyclo(2.2.1)hept-5-ene-2,3-dicarboximide (**1**). ^{1}H-NMR (400 MHz, $CDCl_3$): δ 8.14 (s, 1H, -CNHC-), 6.52 (s, 2H, CH=CH), 5.31 (s, 2H, CHOCH), 2.99 (s, 2H, CH-CH); ^{13}C-NMR (100 MHz, $CDCl_3$): δ 176.2, 136.8, 81.2, 48.9. A solution of **1** (1.0 g, 6 mmol) in 10 mL DMF was added dropwise to a flask containing 1,4-dibromobutane (5.18 g, 24 mmol) and K_2CO_3 (4.14 g, 30 mmol) in DMF (10 mL) over a period of 30 min with stirring. The reaction was further stirred for 12 hours, before removal of solvent by evaporation under reduced pressure. The product (**2**) was purified by flash chromatography on a silica column using a hexane:ethyl acetate (2:1 v:v) eluent system. ^{1}H-NMR (400 MHz, $CDCl_3$): δ 6.51 (s, 2H, CH=CH), 5.26 (s, 2H, CHOCH), 3.51 (t, 2H, NCH_2), 3.41 (t, 2H, CH_2Br), 2.84 (s, 2H, CH-CH), 1.84-1.71 (m, 4H, CH_2-CH_2); ^{13}C-NMR (100 MHz, $CDCl_3$): δ 176.5, 136.8, 81.2, 47.6, 38.1, 33.1, 29.8, 26.4.

Scheme S1. Synthesis of pacRNA.

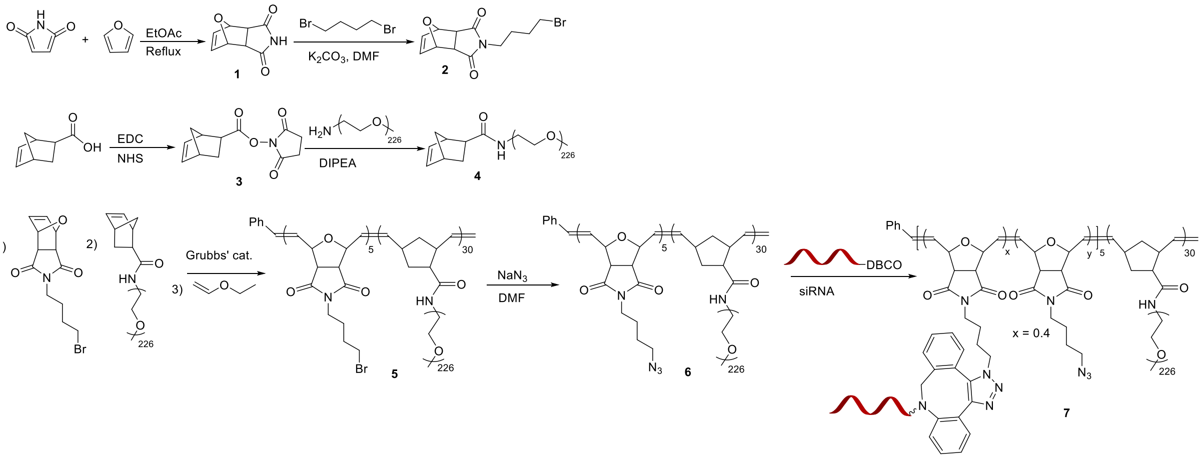

Synthesis of norbornenyl PEG (4). Norbornenyl PEG (**4**) was synthesized in two steps as described previously (*45*). First, the norbornenyl NHS ester was prepared by esterification of norbornenyl carboxylic acid with *N*-hydroxysuccinimide (NHS) using the activating reagent, ethyl(dimethylaminopropyl) carbodiimide (EDC). Then, ω-amine-terminated poly(ethylene glycol) methyl ether (mPEG-NH_2, 1.0 g, 0.1 mmol, M_n = 10 kDa) and norbornenyl NHS ester (30.5 mg, 0.13 mmol) were stirred in a dichloromethane solution (20 mL) containing of *N*,*N*-diisopropyl ethyl amine (DIPEA, 16 mg, 0.1235 mmol) for 6 h at room temperature. This reaction was monitored by MALDI-TOF MS until all mPEG-NH_2 was consumed. The solution was concentrated and precipitated into cold diethyl ether three times, and the final product was dried under vacuum for 48 h.

Synthesis of diblock brush copolymer (6). Modified 2nd generation Grubbs' catalyst was synthesized according to a previously reported method (*46*). A solution of norbornenyl bromide (**2**, 5 equiv.) in deoxygenated dichloromethane was added by gastight syringe into a Schlenk flask under N_2. The solution was cooled to -20 ºC in an ice-salt bath, to which modified Grubbs' catalyst (1 equiv.) in deoxygenated dichloromethane was added via a gastight syringe. The reaction mixture was stirred vigorously for 30 min. Thin-layer chromatography (TLC) confirmed the complete consumption of the

monomer. Then, a solution of **4** (30 equiv.) in deoxygenated dichloromethane was added by gas-tight syringe to the reaction. The reaction mixture was further stirred for 6 h, before addition of several drops of ethyl vinyl ether (EVE) to remove the chain-end catalyst. The mixture was stirred overnight and then precipitated into cold diethyl ether three times. The resulting white solids were dried under high vacuum. Next, the polymer (**5**) was treated with an excess of sodium azide in DMF overnight at room temperature. The resulting solution was dialyzed against Nanopure™ water for 24 h, lyophilized, re-dissolved in Nanopure™ water, and injected into an aqueous GPC for collection of the fractions containing the brush polymer. The final polymer (**6**) was further desalted using a NAP-10 column (G.E. Healthcare, IL, USA). FT-IR confirmed the successful incorporation of azide functionalities. To quantify the number of azide groups per copolymer available for coupling, 5 nmol of the polymer was dissolved in Nanopure™ water (200 µL) and conjugated with alkyne-modified fluorescein (Lumiprobe, 0.207 mg, 500 nmol) using copper catalyst ($CuSO_4 \cdot 5H_2O$, 500 nmol; tris(3-hydroxypropyltriazolylmethyl)amine, THPTA, 600 nmol; sodium ascorbate, 2.5 µmol). The reaction mixture was gently shaken on an Eppendorf Thermomixer at room temperature overnight. Thereafter, the solution was dialyzed against a NaCl solution (0.15 M) using dialysis tubing with a MWCO of 6-8 kDa for 48 h. The UV-Vis absorption of the polymer solution at 491 nm was measured and compared with a standard curve. The number of fluorescein molecules per polymer was calculated based on the known polymer concentration. Approximately 5 fluorescein tags were attached to each diblock brush copolymer.

Synthesis of Cy5 labeled brush polymer. To label the brush polymer for *in vivo* fluorescence tracking, the polymer **6** (15 mg, 50 nmol) and Cy5 alkyne (50 nmol, 50 µL 1 mM DMSO solution) were mixed in Nanopure™ water (2 mL), followed by the addition of the catalyst system ($CuSO_4 \cdot 5H_2O$, 40 nmol; THPTA, 50 nmol; sodium ascorbate, 250 nmol). After 12 hours of stirring, the reaction mixture was dialyzed against Nanopure™ water to remove small molecular residuals, concentrated, and purified by aqueous GPC fractionation. The fractions containing the conjugate were collected, combined, desalted, and lyophilized to yield a blue powder. UV-Vis spectroscopy indicated that there was ~0.9 Cy5 dye molecule per brush polymer.

Synthesis and purification of DBCO-SS-RNA and DBCO-RNA. Amine-modified RNA strands were synthesized using standard solid-phase phosphoramidite chemistry. Purified 5' amine-modified RNA (100 nmol) was dissolved in 100 µL of $NaHCO_3$ (0.1 M) buffer, to which 0.5 mg DBCO-SS-NHS or DBCO-NHS was added via 100 µL DMSO solution. The reaction mixture was shaken at 0 ºC overnight. The products (DBCO-SS-RNA or the DBCO-RNA) were purified by reverse-phase HPLC.

Synthesis of pacRNAs ($pacRNA_{Clv}$ and $pacRNA_{NClv}$) and antisense pacDNA. In a typical procedure, azide-functionalized brush copolymers were dissolved in 200 µL Nanopure™ water, to which DBCO-modified RNA or DNA were added (4 equiv. to N_3, 100 µL aqueous solution). The reaction mixtures were shaken gently for 24 h at 45 ºC on an Eppendorf Thermomixer. Thereafter, aqueous GPC was used to isolate the conjugation product from unreacted DNA or RNA. The conjugates (**7**) were desalted using a NAP-10 column and lyophilized to yield a white powder (or green/red/blue powders for fluorescein-, Cy3-, and Cy5-labeled conjugates). To form double-stranded (ds) oligonucleotides, the solid powders were dissolved in phosphate-buffered saline (PBS), followed

by the addition of 1 equiv. of the single-stranded (ss) complementary sequence. The solutions were annealed by heating to 80 ºC and cooled to room temperature in a thermally insulated container over 24 h.

Synthesis of off-on fluorescent reporter pacRNA for intracellular release monitoring. Alkyne-modified dabcyl (quencher) was first synthesized by coupling dabcyl NHS ester and propargylamine via amidation chemistry. The quencher was then reacted with the brush copolymer (**6**) via copper-catalyzed click chemistry (vide supra, 5:1 mol:mol alkyne:N3). The reaction mixture was stirred overnight, dialyzed against Nanopure™, and was further purified by aqueous GPC. The fractions containing the conjugate were collected and desalted by dialysis. The final solution was lyophilized to yield an orange powder. UV-Vis spectroscopy indicated that there was ~4 dabcyl molecules per brush polymer. Next, the remaining N3 groups on the dabcyl-containing brush polymer were reacted with an excess (~2 equiv. to N3) of DBCO-modified RNA (fluorescein-labeled) by gentle shaking on an Eppendorf Thermomixer at 45 ºC for 24 hours. The final product was purified by aqueous GPC, desalted by a NAP-10 column, and lyophilized. UV-vis quantification indicates ~1 RNA strand per brush polymer.

Molecular dynamics (MD) simulation. MARTINI coarse-grained (CG) force-field with explicit water was used for MD simulation of pacRNA (*47*). The force field incorporates four heavy atoms with similar chemical identities into one CG bead, and therefore reduces the freedoms of the molecules needed to calculate. Bonded parameters are defined based upon molecular structure, while non-bonded parameters, including van der Waals and electrostatic forces, are derived from free energy partitioning between polar and organic solvents. The MARTINI version of PEG was developed by Lee et al (*48*). The atomistic to CG mapping is 3:1 for the PEG monomer. This mapping ratio deviates from the standard MARTNI mapping scheme due to the size of PEG monomer. Herein, the PEG monomer is represented by an SN0 particle in the CG force field. The LJ interaction parameters between PEG and water are $\sigma = 0.47$ nm and $\varepsilon = 4.0$ kJ/mol. The MARTINI version of RNA has been recently developed based upon its DNA predecessor (*49*). The tertiary structure of RNA was constrained using an elastic network. Time step of CG MD simulations was set to be 0.010 ps with periodic boundary conditions. The system was controlled using an NPT ensemble. The temperature was maintained at 310 K using Berendsen temperature coupling while the pressure was controlled at 1 atm by the Berendsen pressure rescaling method (*50*). Cutoff distance of van der Waals and short-ranged electrostatic interactions was set at 1.2 nm. Long-ranged electrostatic interactions were not considered. All simulations were performed on high-performing computing clusters using the GROMACS 5.0.5 package (*51*).

Hybridization kinetics. Fluorescein-labeled free RNA, pacRNAs ($\text{pacRNA}_{\text{Clv}}$ and $\text{pacRNA}_{\text{NClv}}$), and Y-shaped PEG (40 kDa)-RNA conjugate were dissolved in PBS buffer (pH = 7.4) at a final RNA concentration of 100 nM. A total of 1 mL solution for each sample was transferred to a fluorescence cuvette. Then, dabcyl-labeled complementary strand or non-complementary dummy strands (2 equiv.) were added via 2 µL of PBS solution into the cuvette and the solution was rapidly mixed with a pipette. The fluorescence of the solution (ex = 490 nm, em = 520 nm) was continuously monitored every 3 seconds for 60 min. The endpoint was determined by adding a large excess (10 equiv.) of the complementary dabcyl-RNA to the mixture, followed by incubation for 2 h. The kinetics plots were normalized to the endpoint determined for each sample, and all measurements were repeated three

times.

Nuclease degradation kinetics. Free RNA, pacRNAs ($pacRNA_{Clv}$ and $pacRNA_{NClv}$), and Y-shaped PEG (40 kDa)-RNA conjugate (1 µM RNA, fluorescein-labeled) were each mixed with their complementary dabcyl-labeled RNA (2 µM) in PBS buffer. The solutions were gently shaken at room temperature overnight. Subsequently, 100 µL of each sample was withdrawn and diluted to 100 nM with assay buffer (50 mM tris-HCl, 50 mM NaCl, and 20 mM $MnCl_2$, pH = 7.5), to which RNase III (0.4 unit/mL) was added and rapidly mixed. The fluorescence of each sample was monitored every 3 seconds (ex = 490 nm, em = 520 nm) for 5 h. The endpoint of each sample was determined by adding a large excess of RNase III (~2 units/mL) to the mixture followed by continued monitoring until no additional increase in fluorescence was observed. The kinetics plots were normalized to the endpoints of each sample, and all measurements were repeated three times.

siRNA release *in vitro*. Conjugates ($pacRNA_{Clv}$ or $pacRNA_{NClv}$, 100 nM) were mixed with 10 mM DTT in 1× PBS at 37 °C for 5 through 240 min. Thereafter, the solutions were subject to agarose gel electrophoresis using 0.5% agarose gel in 0.5× TBE buffer with a running voltage of 120 V. The amount of siRNA released was determined using band densitometry analysis. All experiments were conducted in triplicates and the results were expressed as the average density value with standard deviations.

Cell culture. SKOV3 and HDF cells were cultured in DMEM supplied with 10% fetal bovine serum (FBS), 1% L-glutamine, and 1% antibiotics at 37 °C in a humidified atmosphere containing 5% CO_2. Human SKBR3 cells were cultured in complete RPMI medium (RPMI 1640 containing 10% FBS, 1% L-glutamine, and 1% antibiotics) at 37 ºC in 5 % CO_2.

Cell uptake. Cellular uptake of pacRNAs and controls was evaluated using flow cytometry and confocal laser scanning microscopy. For flow cytometry, cells were seeded in 6-well plates at 2.0×10^5 cells per well in 2 mL complete DMEM and cultured for 24 h at 37 °C with 5% CO_2. Then, Cy3-labeled PO ds RNA, $pacRNA_{Clv}$, $pacRNA_{NClv}$, PS ss RNA, and PS ds RNA (100 nM-2 µM equiv. of siRNA) dissolved in DMEM or RPMI culture medium was added, and cells were further incubated at 37 °C for 4 h. Subsequently, cells were washed with PBS 3× and suspended by treatment with trypsin. Thereafter, 2 mL of PBS was added to each culture well, and the solutions were centrifugated for 5 min (1000 rpm). Cells were then resuspended in 0.5 mL of PBS for flow cytometry analysis on a BD FACS Calibur flow cytometer. Data for 1.0×10^4 gated events were collected. The experiments were carried out in triplicates.

For confocal microscopy, cells were seeded in 24-well glass bottom plates at 1.0×10^5 cells per well and cultured for 24 h in 1 mL complete DMEM for 24 h at 37 °C with 5% CO_2. Then, Cy3-labeled PO ds RNA, $pacRNA_{Clv}$, $pacRNA_{NClv}$, PS ss RNA, and PS ds RNA (1 µM equiv. of RNA) dissolved in DMEM or RPMI culture medium was added and incubated at 37 °C for 4 h. Thereafter, cells were washed with PBS 3× and fixed with 4% paraformaldehyde for 30 min at room temperature, followed by another 3× washing with PBS. Before imaging, the cells were stained with Hoechst 33342 for 10 min. The cells were imaged on an LSM-700 confocal laser scanning microscope (Carl Zeiss Ltd., Cambridge, UK). Imaging settings were kept identical for all samples in each study.

MTT assay. The cytotoxicity of $pacRNA_{Clv}$ and $pacRNA_{NClv}$ was evaluated with the MTT assay against SKOV3 cells. Briefly, SKOV3 cells were seeded into 96-well plates at 1.0×10^4 cells per well in 200 μL DMEM and cultured for 24 h. The cells were then treated with free RNA, $pacRNA_{Clv}$, and $pacRNA_{NClv}$ at varying concentrations of RNA (50 through 1000 nM). Cells treated with vehicle (PBS) were used as a negative control, and Lipofectamine 2000 (Invitrogen Co., CA, USA)-treated cells were used as a positive control. After 48 h of incubation, 20 μL of 5 mg/mL MTT stock solution in PBS was added to each well. The cells were incubated for another 4 h, and the medium containing unreacted MTT was removed carefully. The resulting blue formazan crystals were dissolved in 200 μL per well DMSO, and the absorbances (490 nm) were measured on a BioTek® Synergy™ Neo2 Multi-Mode microplate reader (BioTek Inc., VT, USA).

Hemolysis. A hemoglobin-free RBC (2% w/v) suspension was prepared by repeated centrifugation (2000 rpm for 10 min at 4 °C) and resuspension in ice cold PBS for a total of 3×. After the final resuspension, the concentration of RBCs was adjusted to 2% w/v. Thereafter, samples (free RNA, $pacRNA_{Clv}$, $pacRNA_{NClv}$, and Lipofectamine-complexed RNA, 1 μM equiv. RNA) were dissolved in PBS, added to the RBC suspension in 1:1 (v:v) ratio, and incubated for 1 h at 37 °C. Complete hemolysis was attained using 2% v/v Triton-X, yielding the 100% control value. After incubation, centrifugation (2000 rpm for 10 min at 4 °C) was used to isolate intact RBCs, and the supernatants containing released hemoglobin were transferred to quartz cuvettes for spectrophotometric analysis at 545 nm. Results were expressed as the amount of hemoglobin released as a percentage of total.

Activated partial thromboplastin time. The aPTT assay was performed on a model BFT-2 coagulometer (Siemens, USA) to determine the clotting times for each sample. First, normal human plasma (50 μL) was incubated with aPTT-XL (50 μL, ThermoFisher, MA, USA) at 37 °C for 5 min. Thereafter, controls/samples (up to 60 μM of RNA) were added and the mixtures were further incubated for another 5 min. Finally, $CaCl_2$ (50 μL of 0.025 M) was added to each mixture to initiate the coagulation. The time until clot formation after the addition of $CaCl_2$ was automatically recorded by the coagulometer. All experiments were performed in triplicates.

Histochemical analyses. Following the anti-tumor study, mice were euthanized with CO_2, and tumors and major organs (heart, lung, liver, spleen and kidney) from each group were excised, fixed in 4% paraformaldehyde/PBS for 6 h, and placed into a 30% sucrose/PBS solution overnight at 4 °C. The fixed tissues were paraffin-embedded and cut into 8 μm-thick sections with a cryostat. The sections were then processed with H&E staining. Immunohistochemistry targeting Bcl-2 was also carried out using mouse anti-Bcl-2 primary antibody (1:100 dilution, Invitrogen Co., CA, USA) and goat anti-mouse secondary antibody (1:1000 dilution, ThermoFisher, MA, USA). In addition, TUNEL assay was performed using the In Situ Cell Death Detection Kit (Roche, Switzerland) on sectioned tissues.

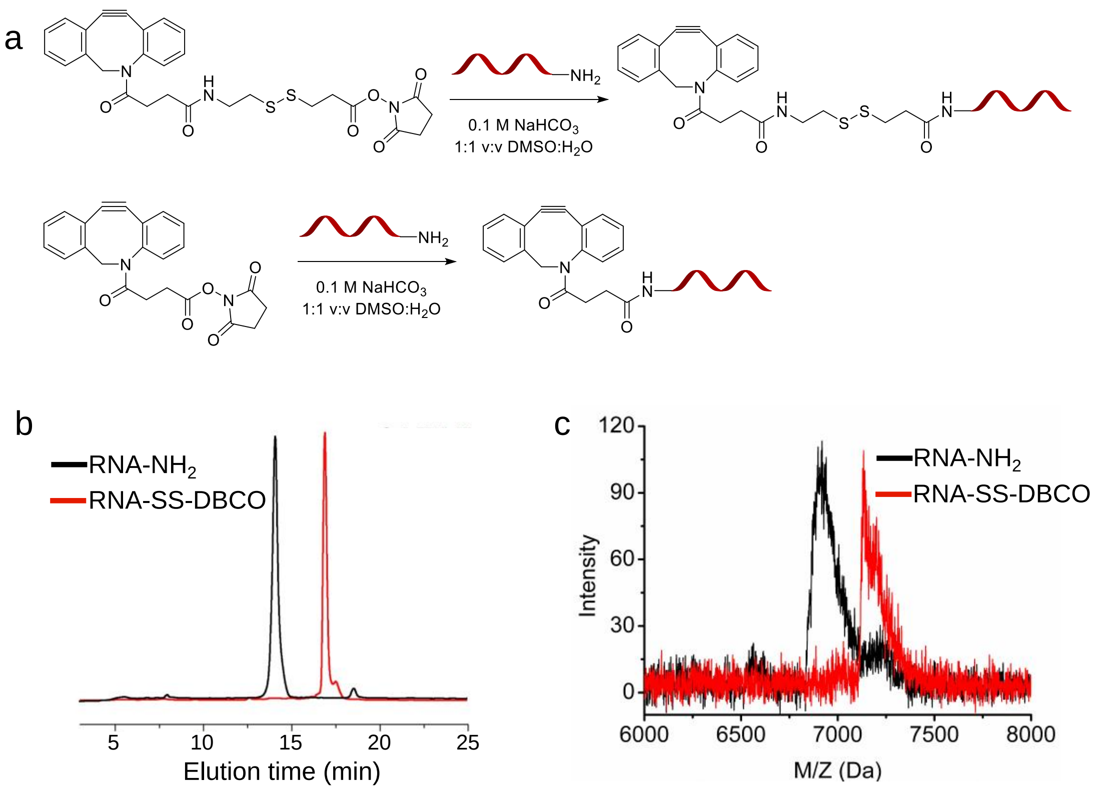

Fig. S1. Synthetic scheme and characterization of dibenzocyclooctyne-modified RNA. Synthetic scheme (**a**) and characterization of DBCO-modified RNA. (**b**) Reverse-phase HPLC chromatograms and (**c**) MALDI-TOF mass spectra of the $RNA\text{-}NH_2$ and RNA-SS-DBCO.

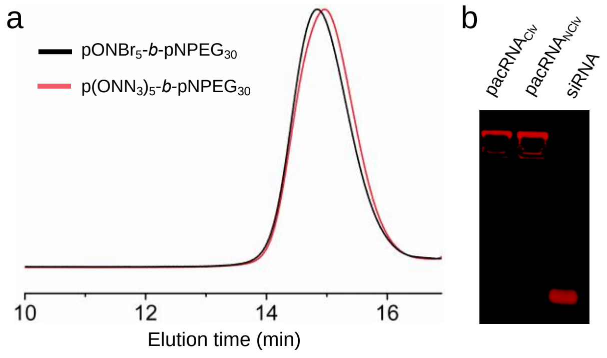

Fig. S2. Additional characterization of brush polymers and pacRNAs. (**a**) DMF GPC chromatograms of the diblock bromide-modified brush $pONBr_5$-*b*-$pNPEG_{30}$ and the corresponding azide-modified brush, $p(ONN_3)_5$-*b*-$pNPEG_{30}$. (**b**) Agarose gel (1%) electrophoresis of Cy3-labeled pacRNAs and siRNA.

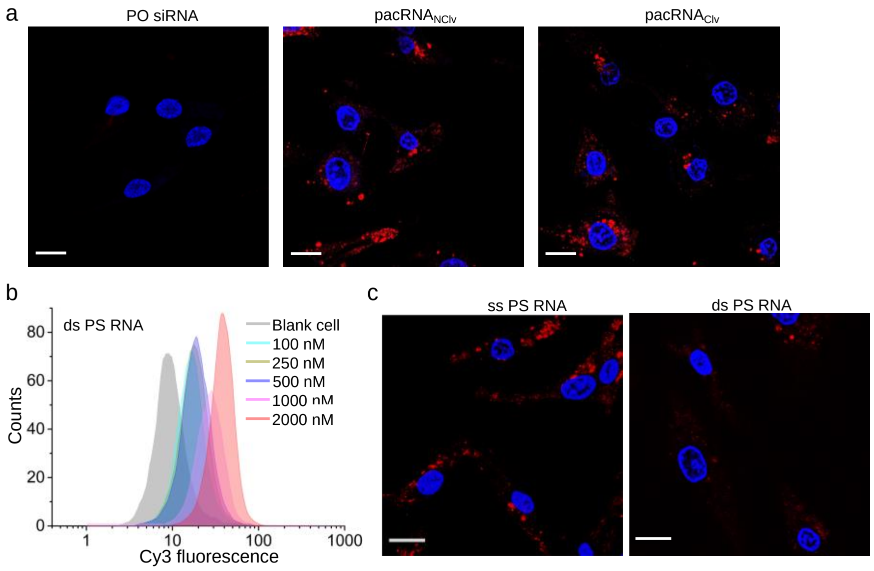

Fig. S3. Cellular uptake of PO siRNA, PS RNA, and pacRNA in SKOV3 cells. (**a**) Representative confocal image of SKOV3 cells treated with Cy3-labeled PO siRNA and pacRNA (red) for 4 h. Cell nuclei were stained with Hoechst 33342 (blue). Imaging settings were kept identical in (a). (**b**) Flow cytometry measurements (total cell counts: 10,000) of SKOV3 cells treated with varying concentrations of ds PS RNA for 4 h. (**c**) Representative confocal images of SKOV3 cells treated with Cy3-labeled ss PS RNA and ds PS RNA for 4 h. Imaging setting were kept identical in (c). Scale bar: 20 µm.

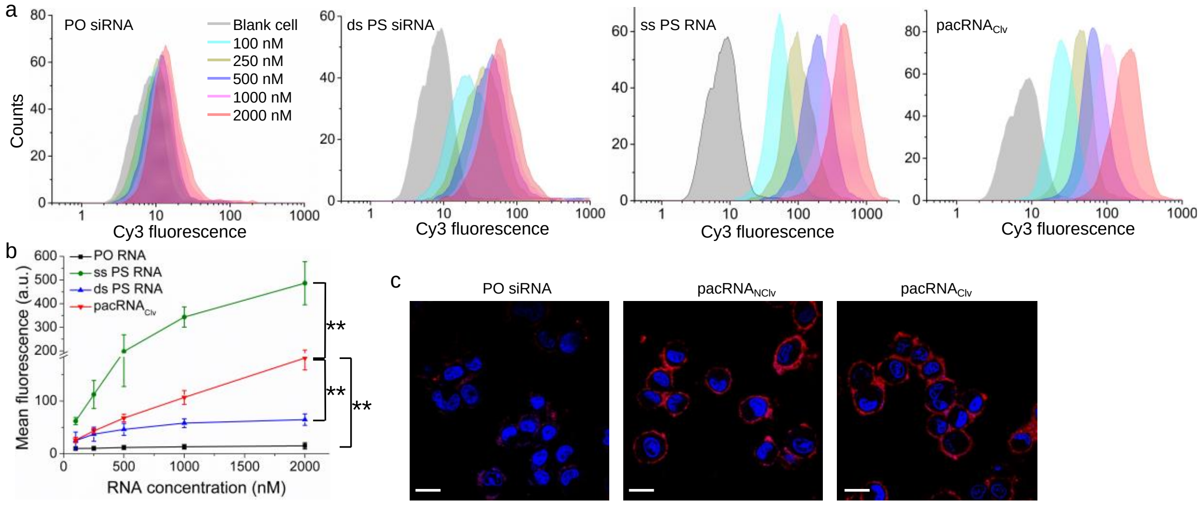

Fig. S4. Cellular uptake of PO siRNA, PS RNA (ss and ds), and pacRNA in SKBR3 cells. (**a**) Flow cytometry measurements (total cell count: 10,000) of SKBR3 cells treated with varying concentrations of samples (100-2000 nM RNA) for 4 h. (**b**) Combined analysis of flow cytometry mean fluorescence showing the different rates of SKBR3 cell uptake of PO siRNA, PS RNA (ss and ds), and pacRNA$_{Clv}$. (**c**) Representative confocal images of SKBR3 cells treated with Cy3-labeled PO siRNA and pacRNAs (red) for 4 h. Cell nuclei were stained with Hoechst 33342 (blue). Imaging setting were kept identical across samples. Scale bar: 20 µm. Error bars refer to the standard deviation of the mean of three independent measurements.

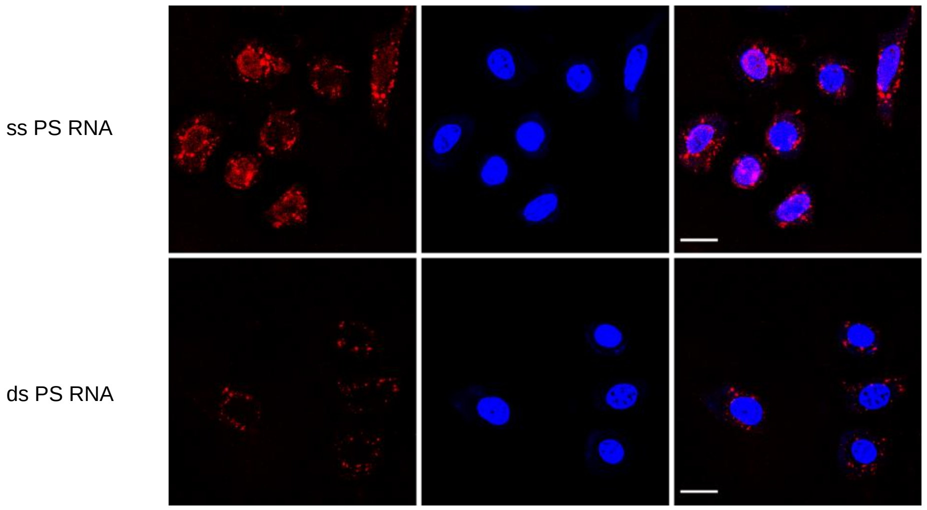

Fig. S5. Representative confocal images of SKBR3 cells treated with Cy3-labeled ss PS RNA or ds PS RNA for 4 h. Cell nuclei were stained with Hoechst 33342 (blue). Scale bar: 20 µm. Imaging setting were kept identical across samples.

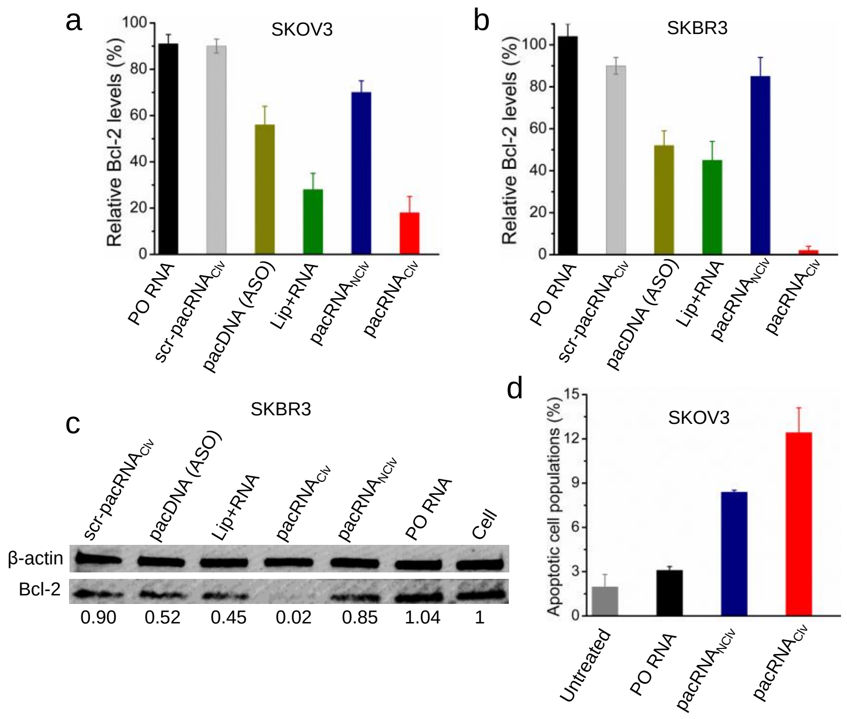

Fig. S6. Bcl-2 down-regulation and cell apoptosis induced by pacRNA. (**a** and **b**) Relative Bcl-2 levels determined by band densitometry analysis of western blots. Bcl-2 expression in SKOV3 and SKBR3 cells were treated with pacRNA and controls (1 µM siRNA) for 6 h in serum-free media, followed by an additional 66 h incubation in fresh, full-serum media. (**c**) A representative western blot of SKBR3 cells treated with pacRNA and controls. (**d**) Apoptotic cell populations following sample treatment as determined by flow cytometry. Error bars refer to the standard deviation of three independent measurements.

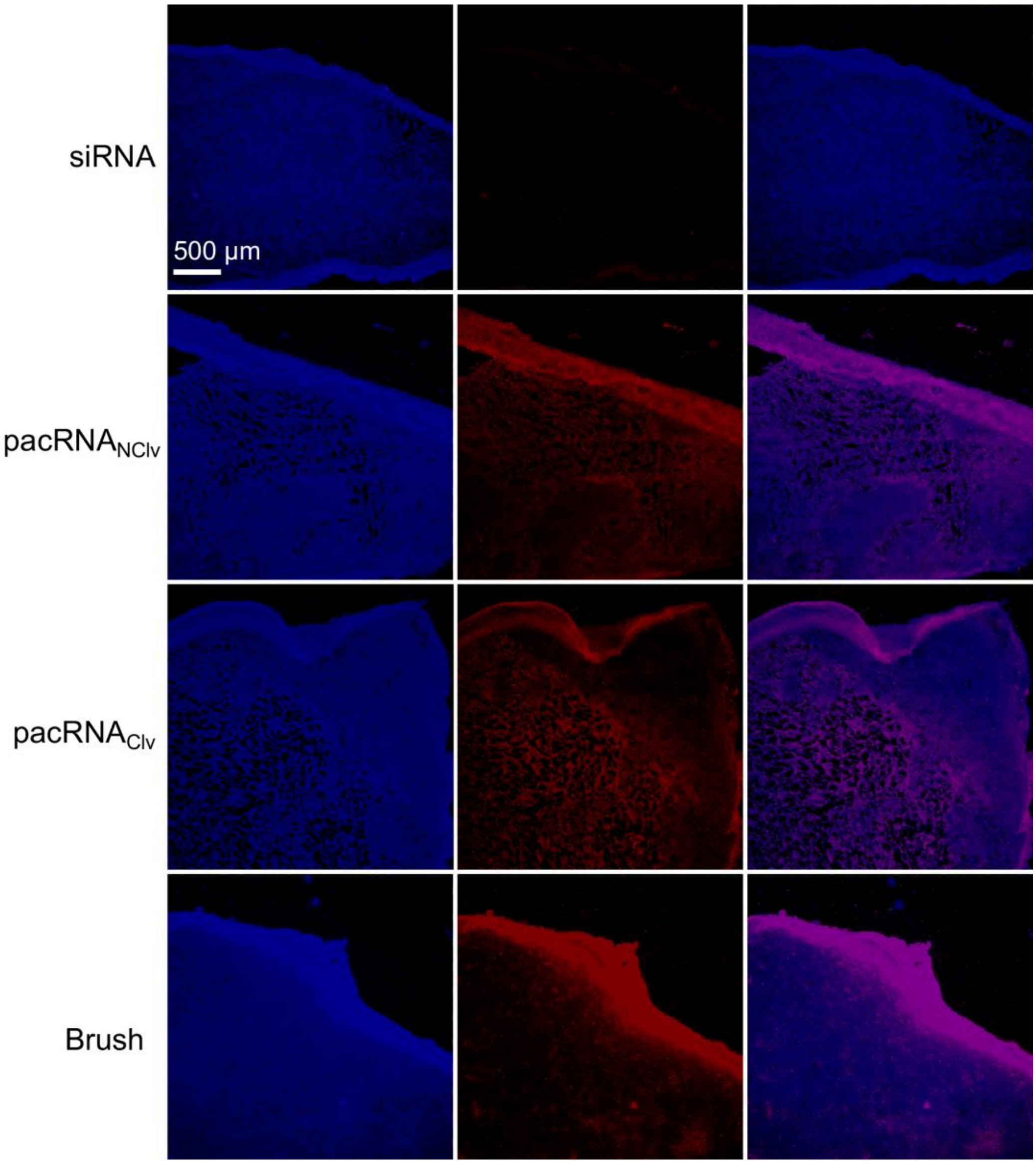

Fig. S7. Fluorescence images of SKOV3 tumor cryosections following intravenous injections of siRNA, pacRNAs, and brush polymers. Red: Cy5-labeled siRNA (for pacRNAs and siRNA) or Cy5.5-labeled brush polymer. Blue: nucleus staining with DAPI.

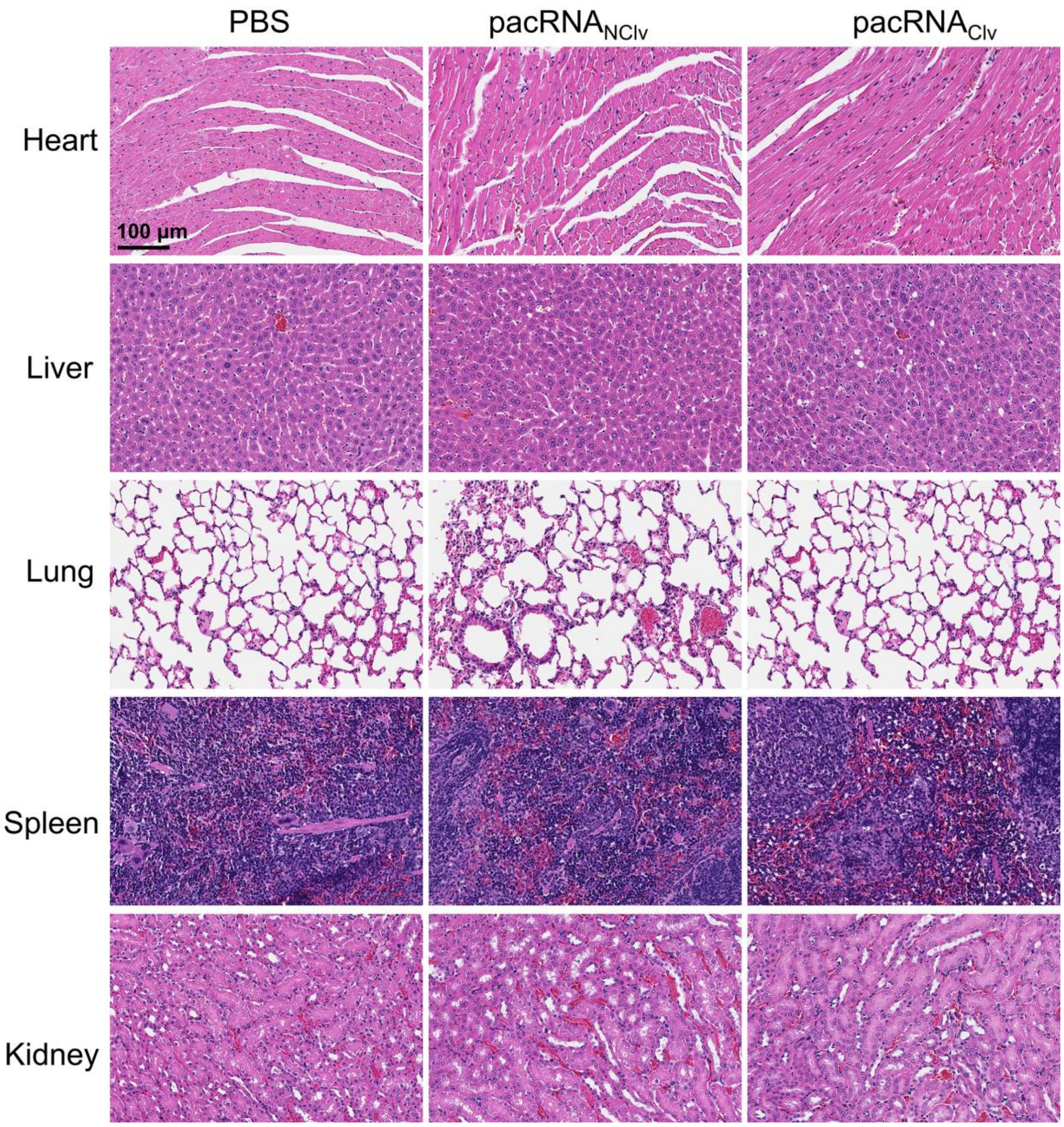

Fig. S8. Microscopic images of H&E-stained sections of various organs from mice after a 32-day treatment period with pacRNAs and PBS showing no apparent histological anomalies.

Table S1. Oligonucleotide sequences.

Amine-modified Bcl-2 antisense	5'-NH_2-CAG CUU AUA AUG GAU GUA C-dTdT-3'
DBCO-modified Bcl-2 antisense	5'-DBCO-CAG CUU AUA AUG GAU GUA C-dTdT-3'
Cy3-labeled and DBCO-modified Bcl-2 antisense	5'-DBCO-CAG CUU AUA AUG GAU GUA C-dTdT-Cy3-3'
Cy3-labeled and amine-modified Bcl-2 antisense	5'-NH_2-CAG CUU AUA AUG GAU GUA C-dTdT-Cy3-3'
Dabcyl-labeled Bcl-2 sense	5'-Dabcyl-GUA CAU CCA UUA UAA GCU G-dTdT-3'
Dabcyl-labeled scrambled sequence	5'-Dabcyl-AUU ACA UAU ACG CCG UUG A-dTdT-3'
Scrambled Bcl-2 antisense	5'-NH_2-AGU AGA GCG UCA UUA UUA C-dTdT-3'
Scrambled Bcl-2 sense	5'-NH_2-GUA AUA AUG ACG CUC UAC U-dTdT-3'
Cy3-labeled PS RNA antisense	5'-rC*rA*rG*rC*rU*rU*rA*rU*rA*rA*rU*rG*rG*rA*rU*rG *rU*rA*rC-Cy3-3'
Dabcyl-labeled PS RNA sense	5'-Dabcyl-rG*rU*rA*rC*rA*rU*rC*rC*rA*rU*rU*rA*rU*rA *rA*rG*rC*rU*rG-3'
Cy5-labeled PS RNA antisense	5'-rC*rA*rG*rC*rU*rU*rA*rU*rA*rA*rU*rG*rG*rA*rU*rG *rU*rA*rC Cy5-3'
PS RNA sense	5'-rG*rU*rA*rC*rA*rU*rC*rC*rA*rU*rU*rA*rU*rA*rA*rG *rC*rU*rG-3'
DNA Bcl-2 antisense strand	5'-DBCO-TTT TCT CCC AGC GTG CGC CAT-3'

Table S2. Plasma pharmacokinetic parameters in C57BL/6 mice.

Sample	$t_{1/2}$ (α) (h)	$t_{1/2}$ (β) (h)	AUC_{∞} (nmol/mL·h)
ds PO siRNA	0.28	0.58	2.8
ds PS siRNA	0.26	0.89	4.1
$pacRNA_{NClv}$	0.28	13.3	53.6
$pacRNA_{Clv}$	0.30	14.8	52.0
Brush polymer	0.25	20.1	88.6
